# Involvement of CXCL1/CXCR2 During Microglia Activation Following Inflammation-Sensitized Hypoxic-Ischemic Brain Injury in Neonatal Rats

**DOI:** 10.3389/fneur.2020.540878

**Published:** 2020-10-06

**Authors:** Meray Serdar, Karina Kempe, Ralf Herrmann, Daniel Picard, Marc Remke, Josephine Herz, Ivo Bendix, Ursula Felderhoff-Müser, Hemmen Sabir

**Affiliations:** ^1^Department of Pediatrics I/Neonatology and Experimental Perinatal Neurosciences, University Hospital Essen, University Duisburg-Essen, Essen, Germany; ^2^Department of Pediatric Oncology, Hematology, and Clinical Immunology, Medical Faculty, Heinrich Heine University, Düsseldorf, Germany; ^3^Department of Neonatology and Pediatric Intensive Care, Children's Hospital University of Bonn, Bonn, Germany; ^4^German Center for Neurodegenerative Diseases (DZNE), Bonn, Germany

**Keywords:** newborn, HIE, brain, infection, inflammation, microglia, chemokine

## Abstract

**Background:** Microglia are key mediators of inflammation during perinatal brain injury. As shown experimentally after inflammation-sensitized hypoxic ischemic (HI) brain injury, microglia are activated into a pro-inflammatory status 24 h after HI involving the NLRP3 inflammasome pathway. The chemokine (C-X-C motif) ligand 1 (CXCL1), and its cognate receptor, CXCR2, have been shown to be involved in NLRP3 activation, although their specific role during perinatal brain injury remains unclear. In this study we investigated the involvement of CXCL1/CXCR2 in brain tissue and microglia and brain tissue after inflammation-sensitized HI brain injury of newborn rats.

**Methods:** Seven-day old Wistar rat pups were either injected with vehicle (NaCl 0.9%) or *E. coli* lipopolysaccharide (LPS), followed by left carotid ligation combined with global hypoxia (8% O_2_ for 50 min). Pups were randomized into four different treatment groups: (1) Sham group (*n* = 21), (2) LPS only group (*n* = 20), (3) Veh/HI group (*n* = 39), and (4) LPS/HI group (*n* = 42). Twenty-four hours post hypoxia transcriptome and gene expression analysis were performed on *ex vivo* isolated microglia cells in our model. Additionally protein expression was analyzed in different brain regions at the same time point.

**Results:** Transcriptome analyses showed a significant microglial upregulation of the chemokine CXCL1 and its receptor CXCR2 in the LPS/HI group compared with the other groups. Gene expression analysis showed a significant upregulation of CXCL1 and NLRP3 in microglia cells after inflammation-sensitized hypoxic-ischemic brain injury. Additionally, protein expression of CXCL1 was significantly upregulated in cortex of male pups from the LPS/HI group.

**Conclusion:** These results indicate that the CXCL1/CXCR2 pathway may be involved during pro-inflammatory microglia activation following inflammation-sensitized hypoxic-ischemic brain injury in neonatal rats. This may lead to new treatment options altering CXCR2 activation early after HI brain injury.

## Introduction

Globally perinatal asphyxia is one of the leading causes of neonatal mortality and long-term morbidity, including mental dysfunctions and cerebral palsy (1). Perinatal asphyxia may lead to neonatal encephalopathy (NE), most likely due to hypoxia-ischemia (HI). To date therapeutic hypothermia (TH) is the standard treatment for hypoxic-ischemic encephalopathy (HIE) with a short therapeutic time-window of 6 h (2). However, almost half of all cooled asphyxiated newborns do not benefit from the treatment (1). As early specific biomarkers are yet not available, early identification of non-responders to TH is not possible. In low- and middle-income countries, where NE rates are significantly higher, the introduction of TH was unsuccessful in decreasing mortality (3). Therefore, the etiology of NE in this population appears to be different. Perinatal infection is an independent risk factor for adverse neurological outcome in term newborns (4). Clinically, it has been shown that infection rates in asphyxiated newborns are significantly higher, compared with the general newborn population (5). Experimentally, we have previously established a newborn animal model of inflammation-sensitized hypoxic-ischemic brain injury in newborn rats. In this model, we found that TH is not neuroprotective (6, 7). This possibly explains the clinical finding, that TH is ineffective in low- and middle-income countries where the prevalence of perinatal infections is higher. Therefore, we need to better understand the pathophysiology underlying inflammation-sensitized hypoxic-ischemic brain injury to explain why TH may be ineffective in this setting.

Hypoxia-ischemia triggers activation of microglia, the resident immune cells of the central nervous system (CNS) (7, 8). In our recent study modeling inflammation-sensitized HI brain injury an upregulation of pro-inflammatory cytokines in microglia 24 h post injury was shown (9). This indicates, that microglia are activated into a pro-inflammatory phenotype early after inflammation-sensitized hypoxia-ischemia. However, the underlying pathways and mechanisms leading to this pro-inflammatory state in the newborn brain are still unknown.

Chemokines are inflammatory mediators, which are released following different brain injuries (inflammation, trauma, hypoxia-ischemia) (10). The chemokine (c-x-c motif) CXCL1 is a chemoattractant for T cells, monocytes, and neutrophils in the brain (11) after binding to its specific CXCR2 receptor. It has been shown, that CXCL1 is essential for the production of reactive oxygen species, which in turn modulate further inflammation (12, 13). One cardinal response to toxic stimuli in the brain is the assembly and activation of inflammasomes. The most defined inflammasome is NLRP3 (nucleotide-binding domain, leucine-rich repeat protein 3), which is activated in neonatal brain injury either due to inflammation (*E.coli* lipopolysaccharide (LPS)) or HI (14, 15). Activation of the NLRP3 inflammasome induces cleavage and therefore activation of interleukin (IL)-18 and IL-1β from its preforms. We have previously shown that NLRP3 gene expression is significantly upregulated in different brain regions (cortex and hippocampus) in our animal model (9). Therefore, we hypothesized that microglia mediated pro-inflammatory cytokine release may be regulated through the CXCL1-NLRP3 pathway following inflammation-sensitized hypoxic-ischemic brain injury in neonatal rats. As previously shown *in vitro* CXCL1 activates the NLRP3 inflammasome through binding to the CXCR2 receptor (13). Here, we investigated the expression of NLRP3 and CXCL1 in microglia and brain tissue in our animal model, aiming to confirm the involvement of CXCL1/CXCR2 in our animal model which may lead to new treatment options for inflammation-sensitized HI brain injury.

## Materials and Methods

### Animals and Experimental Procedure

All animal experiments were performed in accordance to the Animal Research: Reporting of *in vivo* Experiments (ARRIVE) guidelines with government approval by the State Agency of Nature, Environment and Consumer Protection North Rhine-Westphalia, Germany. Seven-day-old (P7) Wistar rat pups of both genders were used in all our experiments. All pups were kept at the central animal laboratory of the University Hospital Essen, Germany with 12:12 dark: light cycle at an environmental temperature 21°C with food and water *ad libitum*. As previously described, all animals were randomized across litter gender and observers blinded to the different treatments (6, 7, 9, 16).

**Figure 1:**
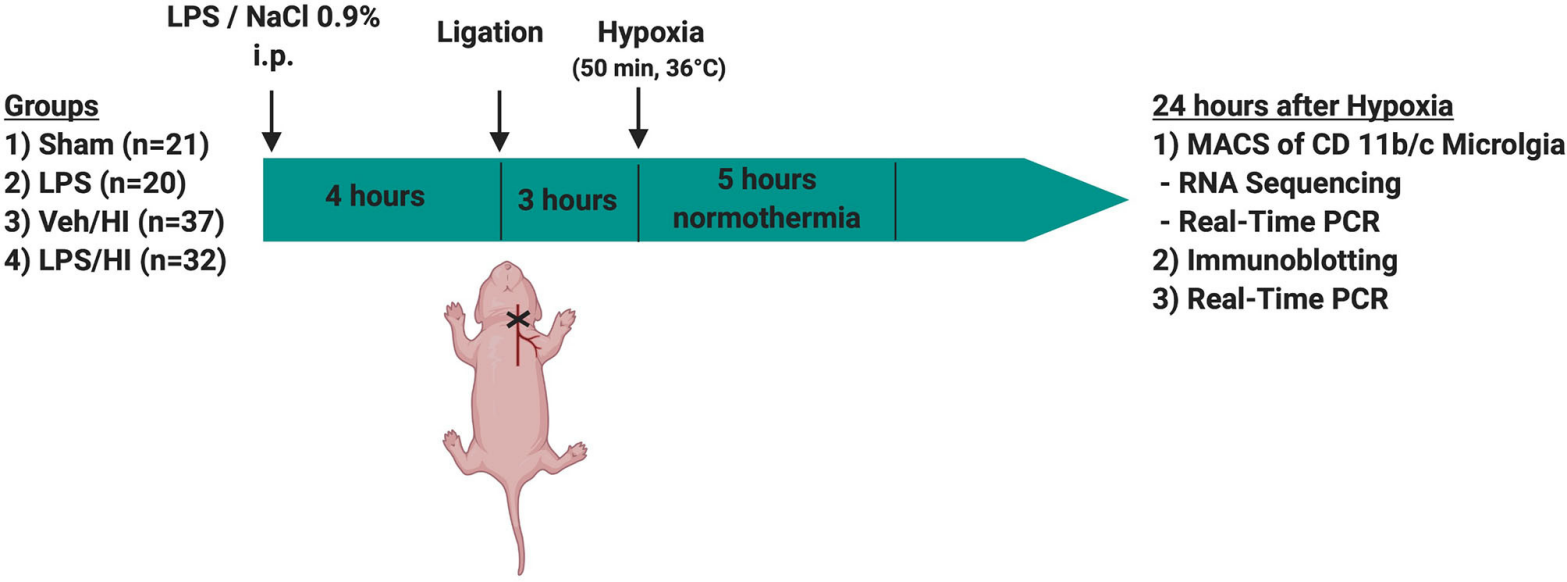
Experimental time course. 7 day old rats (P7) were randomized in 4 different treatment groups. Rats were either injected with NaCl 0.9% or LPS intraperitonally (i.p.) 4 h prior to unilateral ligation of the common carotid artery. Following ligation, rats were exposed to hypoxia (8% O_2_) for 50 min before being treated with normothermia (37°C) for 5 h and thereafter returned to their dam. Twenty-four hours after hypoxia brains were extracted and either used for MACS or Immunoblotting.

Temperature during handling and experimental procedures was monitored in sentinel pups not further allocated to the different treatment groups. All rat pups were kept on a servo-controlled mat (CritiCool, MTRE, Yavne, Israel) during separation from their dams, controlled by the sentinel pup via a rectal temperature probe (IT-21, Physitemp Instruments, Clifton, NJ, United States) continuously maintaining nesting temperature of P7 rats (17) or treatment temperatures during experiments (see below). In our experimental setup, four groups were included: (1) sham group, underwent sham surgery (incision of the neck under isoflurane anesthesia (2% isoflurane) without further operation) (*n* = 21), (2) LPS group, received an intraperitoneal injection (i.p.) of lipopolysaccharide solution (LPS) (*Escherichia coli* lipopolysaccharide O55:B5, Sigma; 0.1 mg/kg) and underwent a sham surgery (*n* = 20), (3) Veh/ HI group, received an injection of 0.9% NaCl, underwent left sided ligation of the common carotid artery under isoflurane anesthesia and were thereafter exposed to hypoxia (8% O_2_) for 50 min at a rectal temperature (T_rectal_) of 36°C—resulting in a mild HI injury as previously described (9) (*n* = 39) and (4) LPS/ HI group, received injection of 0.1 mg/kg LPS and treated with unilateral ligation and exposed to hypoxia as above (*n* = 42). Immediately after the HI insult, pups were kept at T_rectal_ of 37°C for 5 h, as in our previous studies (6–9, 17). After the treatment period, pups were immediately returned to their dam. At 24 h post-HI/sham period, all animals were sedated with chloralhydrate, decapitated, and brain tissue removed, dissected into regions of interest and frozen in liquid nitrogen (Figure 1, Table 1).

**Table 1:** Total number of animals used in this study.

**Experimental group**	**CD11 b/c sorted Microglia RNA [n (m:f)]**		**Whole brain protein [n (m:f)]**	**Whole brain RNA [n (m:f)]**
	**RNA sequencing**	**Real time PCR**		
SHAM	5 (3: 2)	4 (2:2)	7 (4:3)	5 (3: 2)
LPS	5 (3: 2)	4 (2:2)	5 (4:1)	6 (4: 2)
Veh/ HI	10 (3: 7)	8 (5:3)	9 (4:5)	10 (3: 7)
LPS/ HI	8 (3: 5)	8 (5:3)	8 (4:4)	8 (3: 5)

*A total of 110 rats were used in this study. For each analysis rats were randomized between litter and gender (m = male, f = female). 52 animals were used for magnetic cell sorting, 29 animals for whole brain protein analyses and 29 animals were used for whole brain RNA analyses*.

### Magnetic Activated Cell Sorting (MACS) of CD11 b/c Positive Microglia

Twenty four hours after hypoxia-ischemia or sham-operation CD11b/c positive microglia were isolated via magnetic activated cell sorting according to our previous study (9). In groups (1) and (2) entire brains (including ipsi-/contralateral hemispheres) were used for analysis (*n* = 9 per group), while in groups (3) and (4) ipsilateral hemispheres were pooled to get a workable concentration of microglia (*n* = 18 animals in group (3) and *n* = 16 animals in group (4) with 2 hemispheres pooled per sample). The brain tissues were mechanically and enzymatically dissociated by using the Neural tissue dissociation kit by Miltenyi Biotech, followed by myelin removal according the manufacturer's instructions (Miltenyi Biotech, Bergisch Gladbach, Germany). Afterwards the cell suspension was incubated with anti-CD11b/c coupled microbeads followed by magnetic separation of CD11b/c positive microglia. The purity of magnetically sorted microglia was confirmed with immunocytochemistry for the microglia marker Iba1 (data not shown). Microglia were used for all further analyses, except of immunoblotting.

### RNA Sequencing and Gene Set Analysis

We used *n* = 5 animals in groups (1) and (2), whereas, we used pooled ipsilateral hemispheres of two animals in groups (3) (*n* = 10) and (4) (*n* = 8) to get a workable concentration. RNA was isolated using Trizol (Thermo Scientific, Germany) and 500 ng total RNA was processed using the TruSeq RNA Sample Preparation v2 Kit (low-throughput protocol; Illumina, San Diego, USA) to prepare the barcoded libraries. Libraries were validated and quantified using DNA 1000 and high-sensitivity chips on a Bioanalyzer (Agilent, Boeblingen, Germany); 7.5 pM denatured libraries were used as input into cBot (Illumina), followed by deep sequencing using HiSeq 2500 (Illumina) for 101 cycles, with an additional seven cycles for index reading. Fastq files were imported into Partek Flow (Partek Incorporated, Missouri, USA). Quality analysis and quality control were performed on all reads to assess read quality and to determine the amount of trimming required (both ends: 13 bases 5'and 1 base 3'). Trimmed reads were aligned against the rn6 genome using the STAR v2.4.1d aligner. Unaligned reads were further processed using Bowtie 2 v2.2.5 aligner. Aligned reads were combined before quantifying the expression against the ENSEMBL (release 84) database by the Partek Expectation-Maximization algorithm using the counts per million normalization. Genes with missing values and with a mean expression less than one were filtered out. Finally, statistical gene set analysis was performed using a *t*-test to determine differential expression at the gene level (*p* < 0.05, fold change ±2). Partek flow default settings were used in all analyses.

### Real-Time PCR

The RNA of *ex vivo* isolated microglia was isolated by using the RNeasy mini kit from Qiagen according to the distributed instructions. First strand complementary DNA was synthesized using the total RNA of the cells and TaqMan reverse transcription reagents (Applied Biosystems/ Thermo Fisher Scientific, United States). Analysis was performed 24 h after HI injury. We used *n* = 4 animals in groups (1) and (2), whereas we used pooled ipsilateral hemispheres of two animals in groups (3) and (4) (*n* = 8 animals) to get a workable concentration. Additionally, RNA of whole brains was isolated by using the RNeasy mini kit from Qiagen according to the distributed instructions and following procedures as before, for modulation of CXCL1 expression. We used *n* = 5 animals in group (1), *n* = 6 animals in group (2), *n* = 10 animals in group (3), and *n* = 8 animals in group (4). Animals of both genders were used. The expression of the inflammasome cryopyrin (NLRP3) (RN04244620_m1; Life Technologies, Germany) and the chemokine (C-X-C) ligand (Rn00578225_m1; Life Technologies, Germanys) was analyzed. As reference gene we used the Beta-2-microtubuline (B2M, Rn00560865_m1; Life technologies, Germany). Measurements were performed in duplicates and repeated two times for each sample. Target gene expression was quantified according to the 2^ΔΔ^CT method (18). Sham-operated animals served as reference group, as results were normalized to the sham group.

### Immunoblotting

For Western-Blot analysis we used *n* = 29 animals [*n* = 7 in group (1), *n* = 5 in group (2), *n* = 9 in group (3), *n* = 8 in group (4)]. Pups were transcardially perfused with PBS and brain regions were prepared using a standard matrix for uniformity as previously described (9) (ASI Instruments Inc., Warren, MI, United States). We used whole cortex, hippocampus and thalamus for further analyses. Western-Blotting was performed as previously described (16, 19), with adaptions in epitope detection. 12.5% gel membranes were loaded with 40 μg of each sample per lane and were blocked with 5% non-fat dry milk in Tris-buffered saline, 0.1% Tween-20 (TBST, Sigma Aldrich, USA) and incubated overnight (4°C) with the following primary antibodies: polyclonal goat anti C-X-C motif chemokine 1 (1:1,000, Thermo Fisher, Germany, catalog number PA5-86508), polyclonal rabbit anti-NLRP3 (1: 5,000, Abcam, Germany, catalog number ab214185), polyclonal rabbit CXCR2 (1:5,000, Abcam, Germany, catalog number ab65968), and polyclonal mouse anti β-Actin antibody (1:20,000, Sigma, Germany). Horseradish peroxidase-conjugated secondary anti mouse (1:5,000) or anti-rabbit (1:2000, both DAKO, Denmark) antibodies were used. All antibodies were diluted in 5% non-fat dry milk in TBST. Antibody binding was detected by using enhanced chemiluminescence (GE Healthcare Life Sciences, Germany). For visualization and densitometric analysis, ChemiDoc™ XRS+ imaging system and ImageLab software (Bio-Rad, Germany) were used.

### Statistical Analysis

Graphical data are presented as median values with boxplots including the 25% and the 75% percentile. Data were analyzed using GraphPad Prism 6 (GraphPad Software, United States). Differences between groups were determined by one-way analysis of variance (one-way ANOVA) followed by Bonferroni *post hoc* test for multiple comparison. *p*-values < 0.05 were considered as statistically significant.

## Results

In total 2 experiments were performed using a 4-group design, as stated above. Out of the 122 animals used in our 4-group design, mortality was highest in the LPS/HI group. In total 12 animals died during hypoxia, 2/39 from group (3) and 10/42 from group (4), leaving 110 rat pups for further analysis. The high mortality in the LPS/HI group has been expected and reported in our previous studies (6, 7, 9).

### Genome-wide Profiling of Pre-sensitized CD11 b/c Sorted Microglia 24 h After Inflammation-Sensitized Hypoxia Ischemia

**Figure 2:**
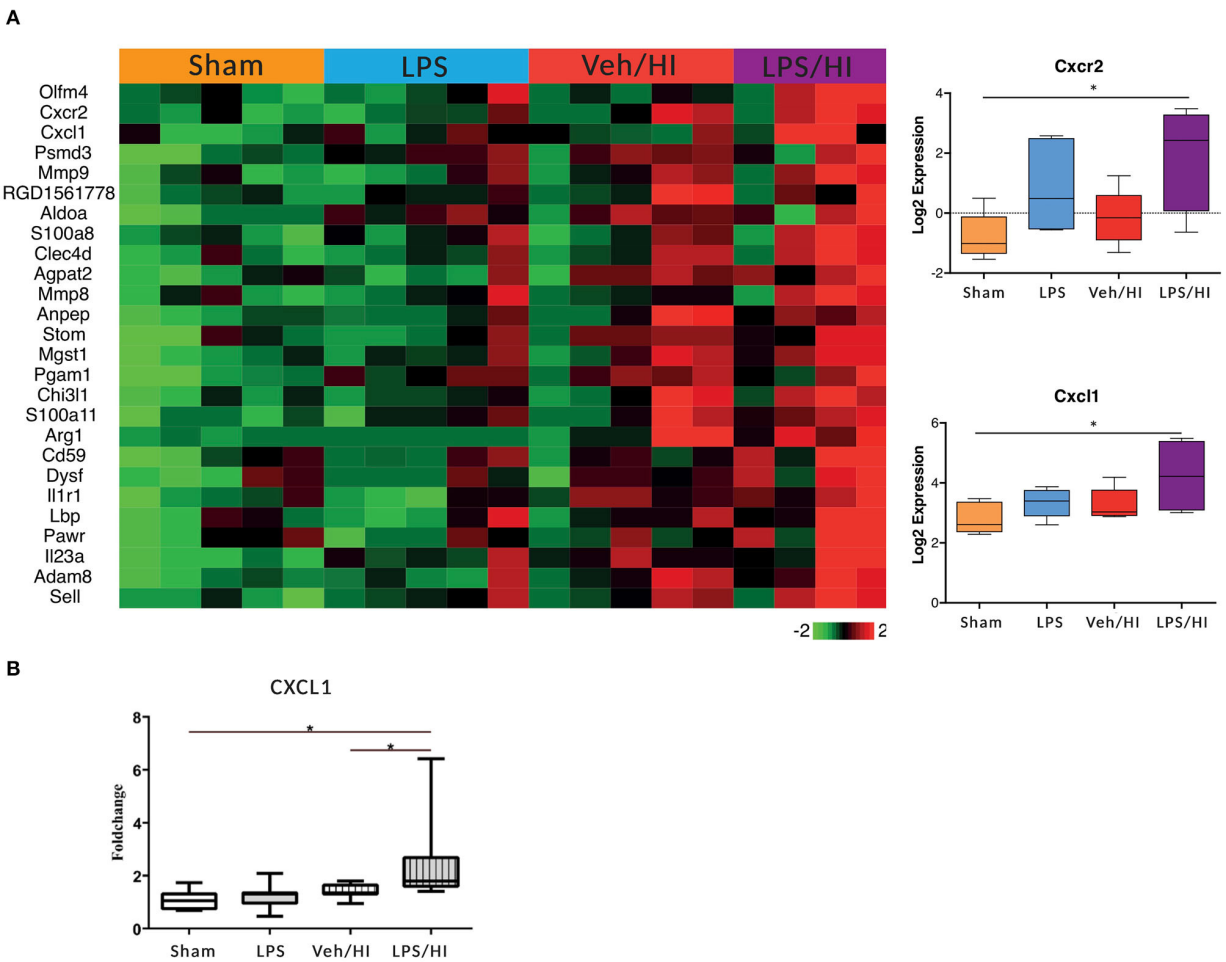
Significant upregulation of several genes especially CXCL1/CXCR2 in inflammation-sensitized HI injury. Twenty-four hours after hypoxia CD11 b/c sorted microglia were used for transcriptome-analysis (RNA-sequencing). The most prominent 26 genes of neutrophil migration and neutrophil degranulation are depicted in a heatmap of each individual group. CXCL1 (ligand to CXCR2) and CXCR2 were significantly upregulated after inflammation-sensitized hypoxic ischemic-brain injury **(A)**. Modulation of CXCL1 expression was confirmed via real time PCR and revealed a significant upregulation after inflammation-sensitized hypoxic ischemic-brain injury **(B)**. *n* = 5 animals in Sham and LPS groups (whole brains), *n* = 10 animals in Veh/HI group and *n* = 8 animals in LPS/HI group (2 pooled ipsilateral brain hemispheres per sample). *p < 0.05.

In our previous study we showed that microglia polarize into a pro-inflammatory phenotype 24 h post HI after pre-exposure to LPS (9). To further investigate this pro-inflammatory polarization, we performed a transcriptomic profiling of microglia cells in our model 24 h post HI. After RNAseq we focused on gene sets of neutrophil migration and neutrophil degranulation, as this clustered gene set showed significant differences in the LPS/HI group compared with all other groups. Also, they are strongly associated with microglia (20). We used a total of 26 genes of interest, as illustrated in a heatmap (Figure 2). We observed a clear upregulation of the CXCR2 and CXCL1 genes in the LPS sensitized HI group compared with the other groups, especially compared with the control group. CXCR2 is a chemokine receptor, which is stimulated by the chemokine CXCL1. Based on the findings and documented results of our previous study (9), we proposed an activation of the inflammasome NLRP3 through a stimulation of the CXCR2 receptor. To verify this hypothesis, we quantified the level of gene expression of the chemokine CXCL1 in CD11b/c positive microglia via RT-PCR, as CXCL1 gene regulation was also significantly upregulated in the LPS/HI group. We determined a significant upregulation of CXCL1 in the LPS/HI group compared with the sham group (*p* = 0.0008) and LPS group (*p* = 0.0182) using RT-PCR.

### Activation of the Inflammasome NLRP3 in Microglia After LPS-Sensitized Hypoxic-Ischemic Brain Injury

**Figure 3:**
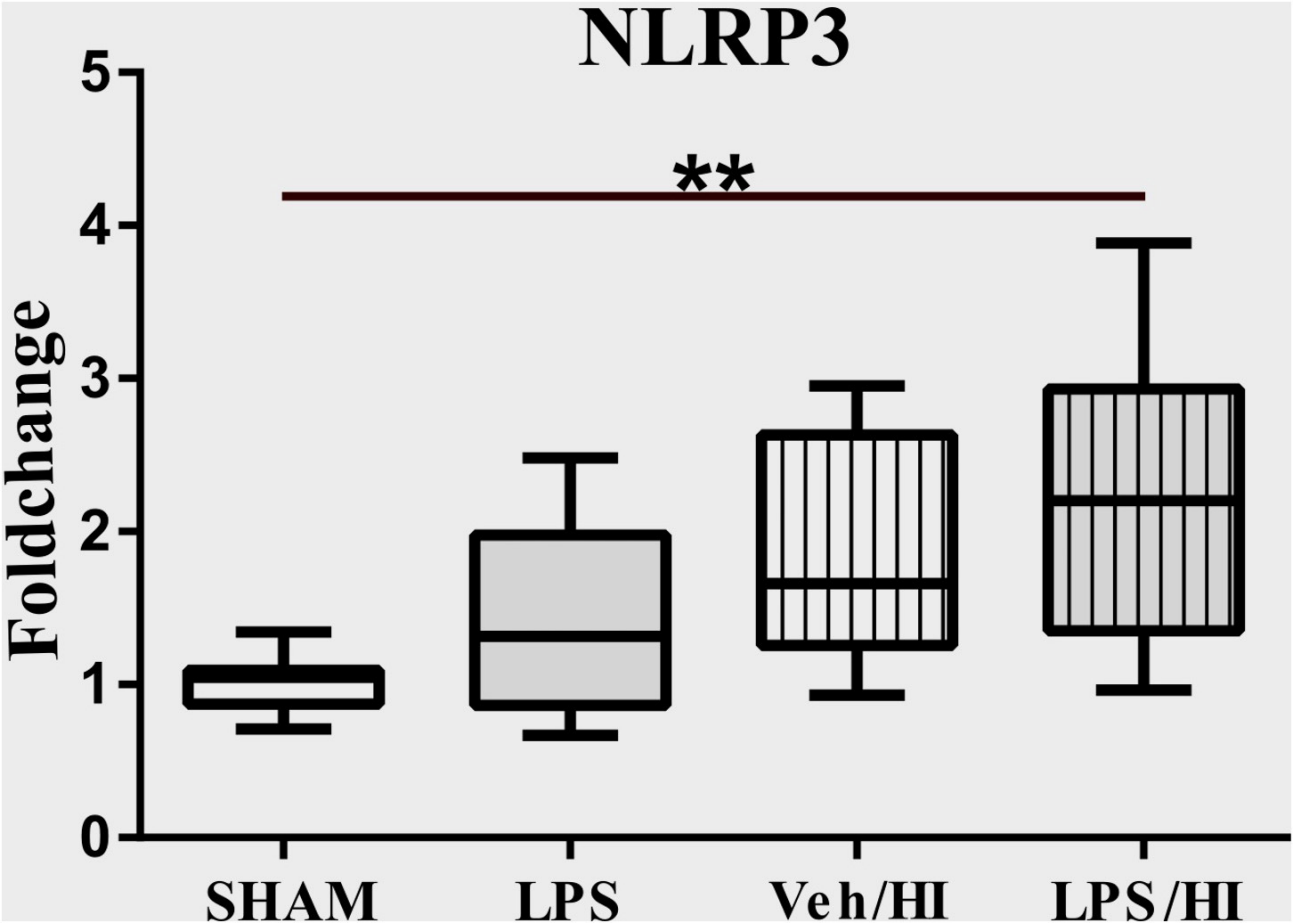
LPS-sensitized hypoxic-ischemic brain injury induces an upregulation of the NLRP3 inflammasome in microglia. Twenty-four hours after HI magnetic sorted microglia were analyzed for the NLRP3-expression via real time PCR. *n* = 4 animals in Sham and LPS groups (whole brains), *n* = 8 animals in Veh/HI and LPS/HI groups (2 pooled ipsilateral brain hemispheres per sample). **p < 0.01.

The NLRP3 inflammasome induces a cleavage of IL-18 and IL-1β, leading to further inflammation by microglia (21). We have previously shown an upregulation of the NLRP3 gene expression in hippocampus and cortex of the LPS/HI group compared with the other three groups (9). However, the modulation of NLRP3 had not been assessed on a cellular level in sorted CD11b/c microglia yet. Therefore, we analyzed the expression of NLRP3 in microglia via RT-PCR and determined a significantly increased expression in the LPS/HI group compared with the Sham group (*p* = 0.0031) (Figure 3).

### Inflammation Sensitized Hypoxia-Ischemia Increases the CXCL1 Protein-Expression in Cortex of Male Pups

**Figure 4:**
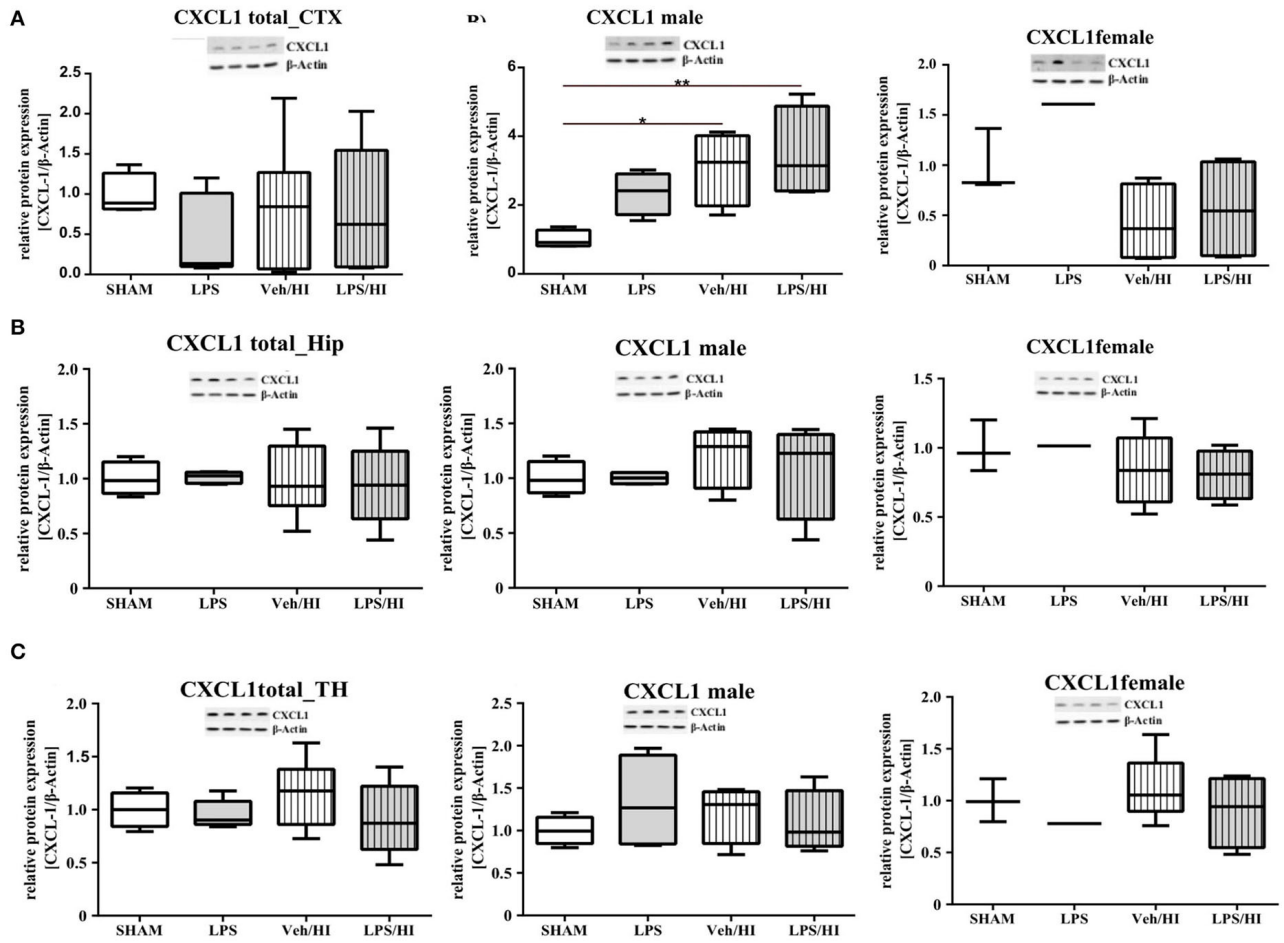
CXCL1 protein expression in different brain regions after inflammation-sensitized hypoxic-ischemic brain injury. Twenty-four hours after hypoxia-ischemia the expression of CXCL1 protein is not regulated in cortex **(A)**, hippocampus (HIP) **(B)** or thalamus (TH) **(C)** from animals of both genders (Sham *n* = 7, LPS *n* = 5, Veh/HI *n* = 9, LPS *n* = 8). A significant upregulation is determined in cortex of males after inflammation-sensitized hypoxic-ischemic brain injury **(A)**, whereas there was no gender specific upregulation in hippocampus **(B)** or thalamus **(C)** (Sham *n* = 4 males, LPS *n* = 4 males, Veh/HI *n* = 4 males, LPS *n* = 4 males). *p < 0.05, **p < 0.01.

**Figure 5:**
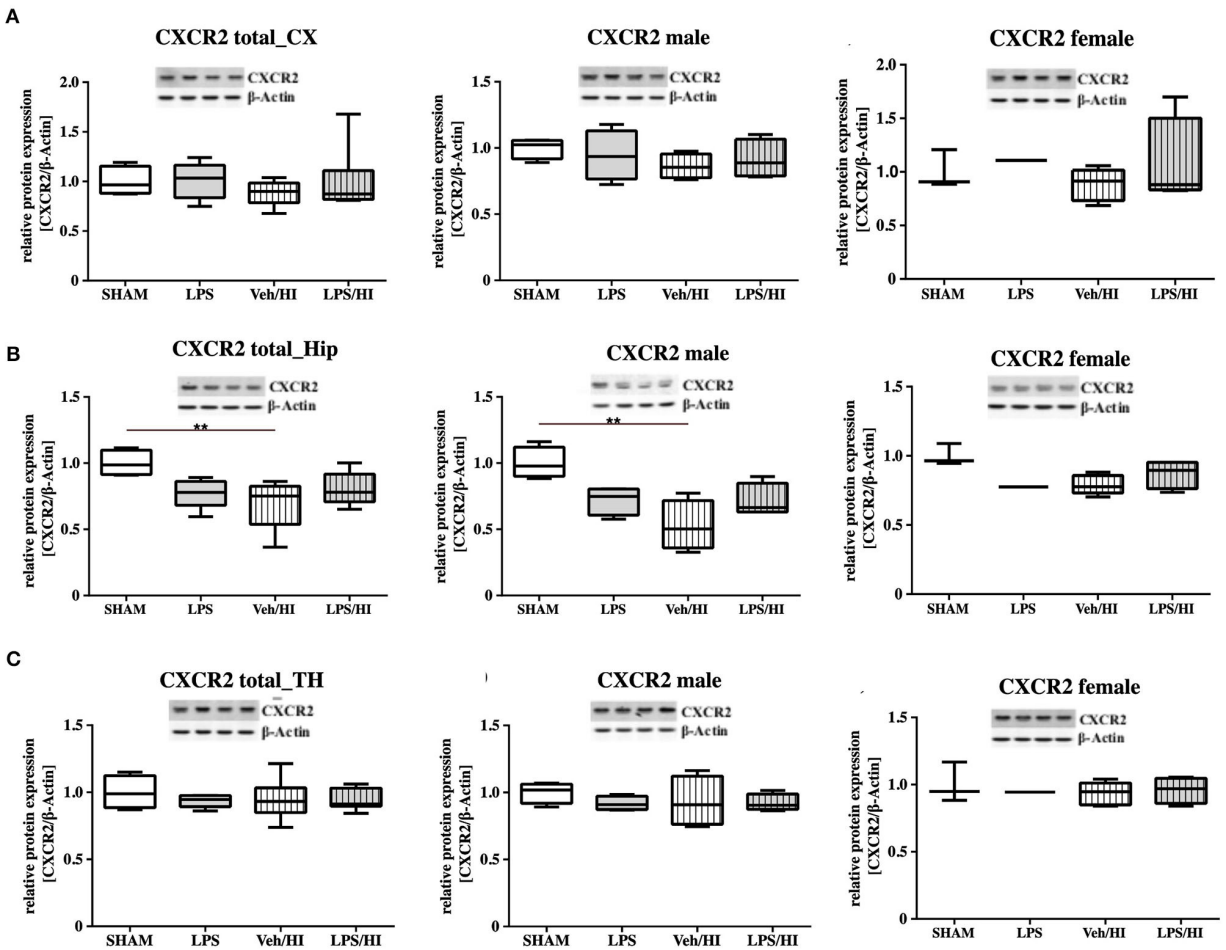
CXCR2 protein expression in different brain regions after inflammation-sensitized hypoxic-ischemic brain injury. Twenty-four hours after hypoxia-ischemia the expression of CXCR2 protein is not regulated in cortex (CX) **(A)**, hippocampus (HIP) **(B)** or thalamus (TH) **(C)** from animals of both genders (Sham *n* = 7, LPS *n* = 5, Veh/HI *n* = 9, LPS *n* = 8). A significant downregulation is determined in hippocampus of males after hypoxic-ischemic brain injury **(B)**, whereas there was no difference in the inflammation-sensitized hypoxic-ischemic brain injury groups (Sham *n* = 4 males, LPS *n* = 4 males, Veh/HI *n* = 4 males, LPS *n* = 4 males). **p < 0.01.

**Figure 6:**
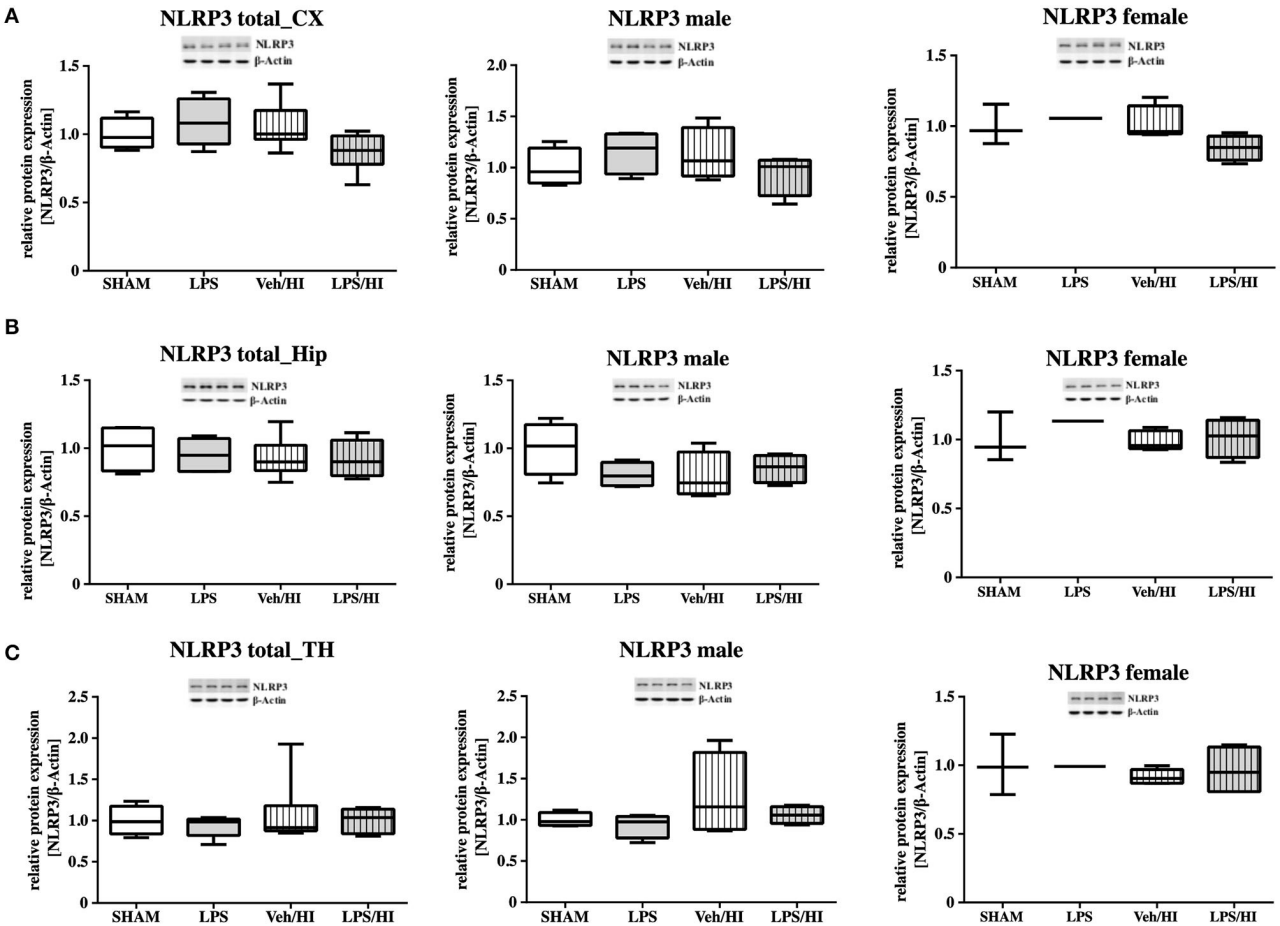
NLRP3 protein expression in different brain regions after inflammation-sensitized hypoxic-ischemic brain injury. Twenty-four hours after hypoxia-ischemia the expression of NLRP3 protein is not regulated in cortex (CX) **(A)**, hippocampus (HIP) **(B)** or thalamus (TH) **(C)** from animals of both genders (Sham *n* = 7, LPS *n* = 5, Veh/HI *n* = 9, LPS *n* = 8). There was no gender differences in any of the groups or analyzed brain regions (Sham *n* = 4 males, LPS *n* = 4 males, Veh/HI *n* = 4 males, LPS *n* = 4 males).

As gene expression levels do not fully represent protein expression levels, we analyzed the protein expression of CXCL1, CXCR2 and NLRP3 in the cortex, hippocampus and thalamus. For CXCL1, we observed no significant modulation in the individual groups as shown in Figure 4. Therefore, we investigated gender differences showing that in LPS-sensitized male pups the protein expression of CXCL1 is significantly increased 24 h after inflammation-sensitized hypoxia-ischemia compared with the Sham group (*p* = 0.0099). However, this gender-specific effect was only seen in the cortex and not in the hippocampus or thalamus. For CXCR2 and NLRP3, we did not observe any differences in the LPS-sensitized hypoxia-ischemia groups within the different brain areas and there was no gender differences (Figures 5, 6).

## Discussion

Up to now TH is the only established standardized neuroprotective treatment for neonatal encephalopathy following perinatal asphyxia. As treatment success might be influenced by different co-morbidities, the subgroup of non-responders to TH needs to be further investigated. In an experimental setup of inflammation-sensitized hypoxic-ischemic brain injury TH has failed to be neuroprotective (6, 7). To improve the understanding for the lack of neuroprotection from TH, we aim to focus on the underlying mechanisms leading to increased inflammation in our established model of inflammation-sensitized HI brain injury. As we have previously shown, microglia are activated into a pro-inflammatory phenotype early after inflammation-sensitized HI (9). In the current study, we show that the chemokine CXCL1 and its receptor (CXCR2) are upregulated and therefore may be involved in the pro-inflammatory response of microglia in our model. In addition, expression of the NLRP3 inflammasome is upregulated in microglia after inflammation-sensitized HI brains. As it has been shown that NLRP3 is regulated by the chemokine CXCL1 and its specific receptor CXCR2, we assume that CXCL1/CXCR2 is involved during the pro-inflammatory activation of microglia and that this activation also involves NLRP3 upregulation in microglia following inflammation-sensitized HI brain injury.

Only fifty percent of cooled asphyxiated newborns benefit from TH, whereas the others develop mild to severe motor-cognitive deficits in later life (1). Perinatal infection can lead to perinatal asphyxia and also neonatal infection rates are much higher in asphyxiated newborns (5, 22). Comparable with a clinical single-center experience (23), TH is not neuroprotective in our established model of inflammation-sensitized HI brain injury (6, 7). Therefore, there is an urgent need of neuroprotective treatments in the setting of inflammation-sensitized HI brain injury, presenting as neonatal encephalopathy. To identify new treatment options the underlying mechanisms, need to be analyzed. As previously shown, brain ischemia triggers a series of events that cause resident microglia to become activated and develop macrophage-like capabilities (24, 25). Microglia activation can be defined by two different phenotypes; a pro-inflammatory phenotype indicated as M1 and an anti-inflammatory phenotype indicated as M2. In the context of neonatal brain injury, it has already been shown that M1/M2 specific genes are upregulated in a time dependent manner (26) and that early M1 activation might be crucial in developing neonatal HI brain injury (27, 28). Recently, we determined an early upregulation of pro-inflammatory cytokines in different brain regions (cortex and hippocampus) in our model of LPS-sensitized HI brain injury, mainly produced by activated microglia (9). Additionally, we hypothesized that the NLRP3 inflammasome is involved in the inflammatory response of microglia in our model (9). Inflammasomes are activated during pathogenic stimulation including NLRPs (13, 29, 30). NLRP3, the most intensively studied inflammasome, has been shown to be involved in many neurological diseases in adults, such as multiple sclerosis, Alzheimer's disease and Parkinson's disease (31). It is a caspase-1 activating multi-protein complex regulating the cleavage of the interleukins 18 and 1β, which are associated with pro-inflammatory processes (32). Previously, we detected an activation of the NLRP3 inflammasome 24 h after hypoxia in selected brain regions (cortex and hippocampus) of inflammation-sensitized newborn rats. We believe, that the upregulation of NLRP3 in brains of the LPS/HI group is involved in the activation of M1 microglia, as we detected a microglia specific secretion of IL-1β (9). Therefore, we analyzed the NLRP3 gene expression in CD11b/c sorted microglia of brains from individual groups in the present study. We found an upregulation of NLRP3 gene expression following inflammation-sensitized HI brain injury, suggesting an involvement of the NLRP3 inflammasome as one potential regulatory pathway in LPS-sensitized HI brain injury. Several possible mechanisms activating the NLRP3 inflammasome have been described. The stimulation of Toll-like receptor 4 through LPS triggers the translocation of nuclear factor kappa B into the nucleus and induces the expression of pro-inflammatory genes in microglia involving the NLRP3 inflammasome (33, 34), indicating its role during CNS inflammation. In order to find out which signaling cascades are involved in the pro-inflammatory response of microglia in the LPS/HI group, we investigated the transcriptomic profile of *ex vivo*-isolated microglia from the defined experimental groups. We observed a significant upregulation of different gene clusters. One cluster were gene sets involved in neutrophil migration and neutrophil degranulation. We therefore focused on this set of genes, as neutrophil migration and neutrophil degranulation have been shown to be microglia associated following neonatal brain injury (28). According to our hypothesis that the induced inflammatory process in our model is due to the activation of the NLRP3 inflammasome, we looked at targets which are associated with the activation of the NLRP3 inflammasome. We observed a significant increase of the chemokine receptor CXCR2 in the LPS/HI group compared with the other groups. As previously described in the literature of adult animal models, interaction between CXCL1 and CXCR2 can modulate the activation of the NLRP3 inflammasome (13). However, its function in neonatal brain injury is still unclear and has not been studied under normothermic or hypothermic conditions yet. We investigated the gene expression of the ligand CXCL1 in microglia and showed a significant upregulation in the LPS/HI group comparable with the increased expression of the NLRP3. To clarify whether the modulation of CXCL1 gene expression in the LPS/HI group is also associated with its modulation on protein level, we performed Western-Blot analysis in different brain regions. However, as we performed Western-Blot analysis in brain lysates and not isolated microglia, we determined no different protein regulation between the treatment groups in any of the analyzed brain regions. Region-specific differences in chemokine or inflammasome activation have also been shown by other groups, representing variability in different models. Ystgaard et al. found different distribution of NLRP3 gene upregulation in their newborn mouse model 24 h post hypoxic-ischemic injury, with a significant upregulation in the hippocampus and thalamus (35). Yellowhair et al. showed that CXCL1/CXCR2 signaling contributes to newborn brain inflammation in an *in-vitro* model of preclinical chorioamnionitis and that blocking the CXCR2 receptor reduces neuroinflammation in different white matter regions (36). Interestingly in our study, we found a gender-specific upregulation of CXCL1 protein level in males in the LPS/HI group compared with the Sham group in the cortex (Figure 4). These gender-specific differences might emphasize the findings by Mammun et al., showing inflammatory responses are gender-specific and males are more sensitive to HI brain injury than females (25). This also corresponds to pre-clinical and clinical observations, where males are more sensitive to ischemic insults and have worse outcomes compared to females (37–39). However, this has to be further confirmed in our model.

There are some limitations in our study. First, analyses were performed at a single time point. However, the inflammation process will change over time. Therefore, we need more time-points to understand the time-depended changes in inflammation and to evaluate possible applicable treatments. Second, to analyze if the microglia mediated pro-inflammatory response in the LPS/HI group is regulated specifically through the CXCL1/NLRP3 pathway, we should use a specific antagonist for the CXCR2 receptor, like SB225002, blocking the receptor and the downstream pathway. Furthermore, cell-type specific experimental approaches will be needed to assess the causal relationship between CXCR2 activation and NLRP3 activation and the functional relevance of this pathway for the development of inflammation sensitized hypoxic-ischemic brain injury. Third, although we found gender-specific differences in our western blot results, we were not able to analyze gender differences in our microglia specific analyses, as we used pooled samples of both genders to receive a feasible working concentration of microglia. Therefore, further gender-specific studies need to be performed in our model using microglia, before a gender-specific effect can be assumed. Fourth, we assumed that differences in gene expression would lead to differences in protein expression. However, this is often not the case, as transcription does not automatically lead to translation with the release of proteins (40). Last, we did not include TH in our study. As shown in adult animal models, TH reduces NLRP3 activation and microglia activation after traumatic brain injury (41) or cardiac arrest (42). However, it has been found in a newborn animal study of hypoxic-ischemic encephalopathy, that NLRP3 deficiency increases brain injury (35). Therefore, before we investigate alterations of the CXCR2/NLRP3 pathway under hypothermic conditions, we need to understand the function and role under normothermic conditions.

Our results demonstrate that microglia reveal an early pro-inflammatory response demonstrated by an upregulation of CXCR2, CXCL1 and NLRP3 gene expression following LPS-sensitized HI brain injury in neonatal rats. These findings may contribute to a better understanding of the complex pathophysiology of NE, which is indispensable to develop new treatment options and to improve outcome in asphyxiated newborns, especially in countries with a high prevalence of perinatal infection and associated asphyxia.

## Data Availability Statement

The raw data supporting the conclusions of this article will be made available by the authors, without undue reservation, to any qualified researcher.

## Ethics Statement

The animal study was reviewed and approved by State Agency of Nature, Environment and Consumer Protection North Rhine-Westphalia, Germany.

## Author Contributions

MS and HS have planned and designed the study and have performed the animal experiments. MS, KK, RH, DP, MR, and HS have performed tissue analysis. MS, RH, DP, MR, and HS have analyzed the data. MS, JH, IB, UF-M, and HS have written and corrected the manuscript. All authors contributed to the article and approved the submitted version.

## Conflict of Interest

The authors declare that the research was conducted in the absence of any commercial or financial relationships that could be construed as a potential conflict of interest.
